# Giant Cervical Occipital Lipoma: A Case Report

**DOI:** 10.7759/cureus.63496

**Published:** 2024-06-30

**Authors:** Mohammed Mhand, Abdelhakim Harouachi, Tariq Bouhout, Badr Serji

**Affiliations:** 1 Department of Surgical Oncology, Mohammed VI University Hospital, Régional Oncologue Center, Oujda, MAR; 2 Surgical Oncology, Faculté de Medecine et de Pharmacie Oujda, Oujda, MAR; 3 Department of Surgical Oncology, Oncology Hospital of Oujda, Oujda, MAR; 4 Faculty of Medicine and Pharmacy of Oujda, University Mohamed Premier, Oujda, MAR

**Keywords:** case report, lipoma, occipital, cervical, giant

## Abstract

Lipomas are common, well-circumscribed neoplasms of mesodermal origin, characterized by being slow, painless growths that are mostly subcutaneous, not invasive, and not recurring after surgery. Lipomas are the most prevalent kind of mesenchymal tumor, yet giant lipomas are rare in the cervical region and the occipital area.

We report a 46-year-old female with diabetes insipidus was referred with a giant occipital cervical tumor, which she had noticed for 17 years and which had rarely given her any complaints of compressive symptoms. The clinical assessment indicated a firm, painless, and mobile swelling, which demonstrated features of venous ectasia, and there was no external ulceration. Ultrasonography and MRI of the neck revealed a large, subcutaneous fatty tumor with distinctive echographic features on both modalities, including hyperintense signals on T1 and T2 and no ring enhancement after Gadolinium injection. Due to the mass being smooth, round, and not attached to any structure, the patient underwent surgical enucleation under general anesthesia, resulting in full recovery without complications. Pathology revealed a benign adipose tissue tumor without liposarcoma, and there were no difficulties observed during follow-up for two years.

## Introduction

Lipomas are benign, encapsulated tumors originating from mesenchymal tissue and developing within adipose tissue [1]. They constitute nearly 13% of tumors found in the head and neck region [1]. Clinically, they generally appear as asymptomatic, slow-growing, and painless masses [1,2].

These tumors are predominantly located subcutaneously throughout the head and neck areas. They are non-invasive, and recurrence is rare following surgical removal. Superficial, simple lipomas can persist for many years without causing functional problems and seldom grow to significant sizes [1].

Giant lipomas are categorized as lesions measuring at least 10 cm in one dimension or weighing a minimum of 1000 g [3]. Their occurrence in the occipital and cervical tissues is uncommon [1]. In this report, we discuss our experience with a patient presenting with a giant cervical-occipital lipoma and its surgical management.

## Case presentation

**Figure 1:**
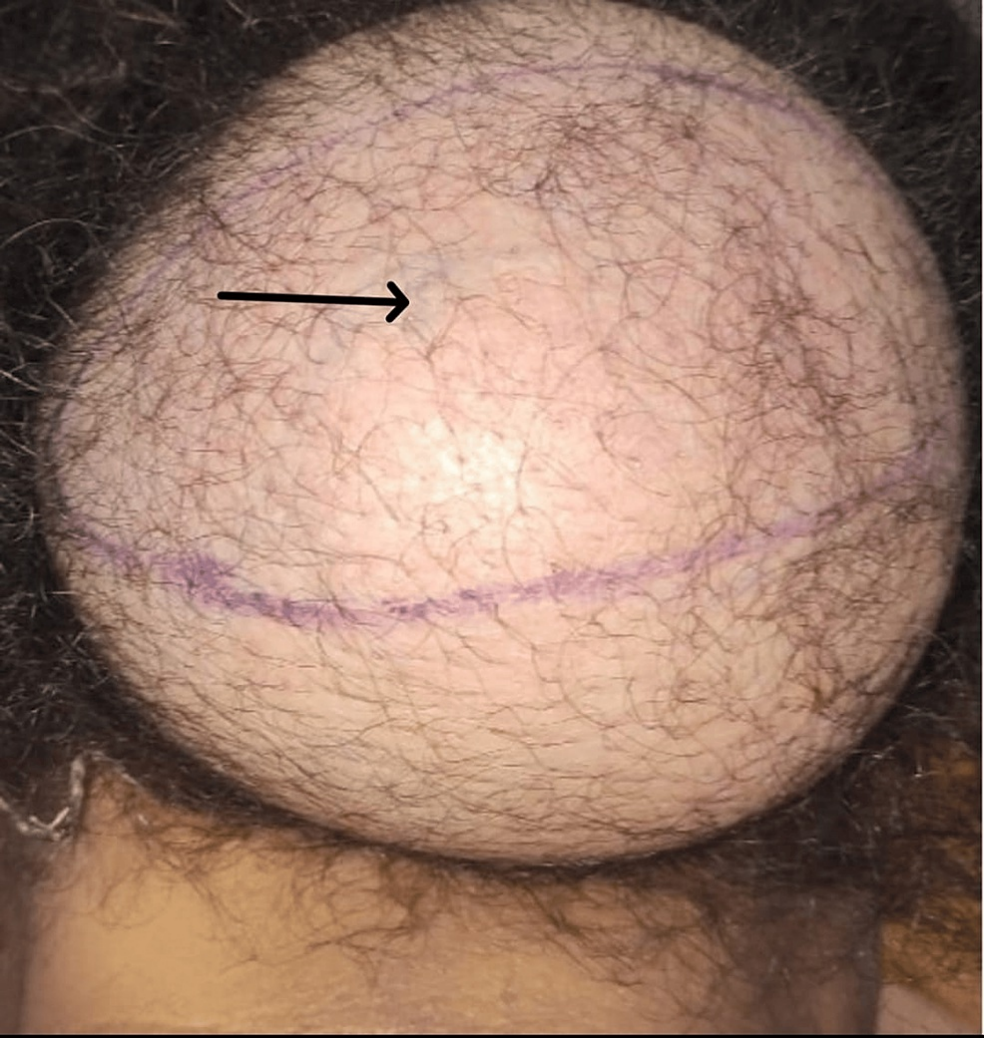
Initial posterior view of cervical occipital region showing the large mass with venous ectasia (black arrow).

A 46-year-old female patient with diabetes on insulin therapy presented with a giant occipito-cervical mass that had been slowly increasing in size over 17 years without causing any compressive syndrome. Clinical examination revealed a huge cervico-occipital mass with venous ectasia and no surface ulceration. The mass was firm in consistency, painless to palpation, and mobile relative to the deeper plane (Figure 1). 

**Figure 2:**
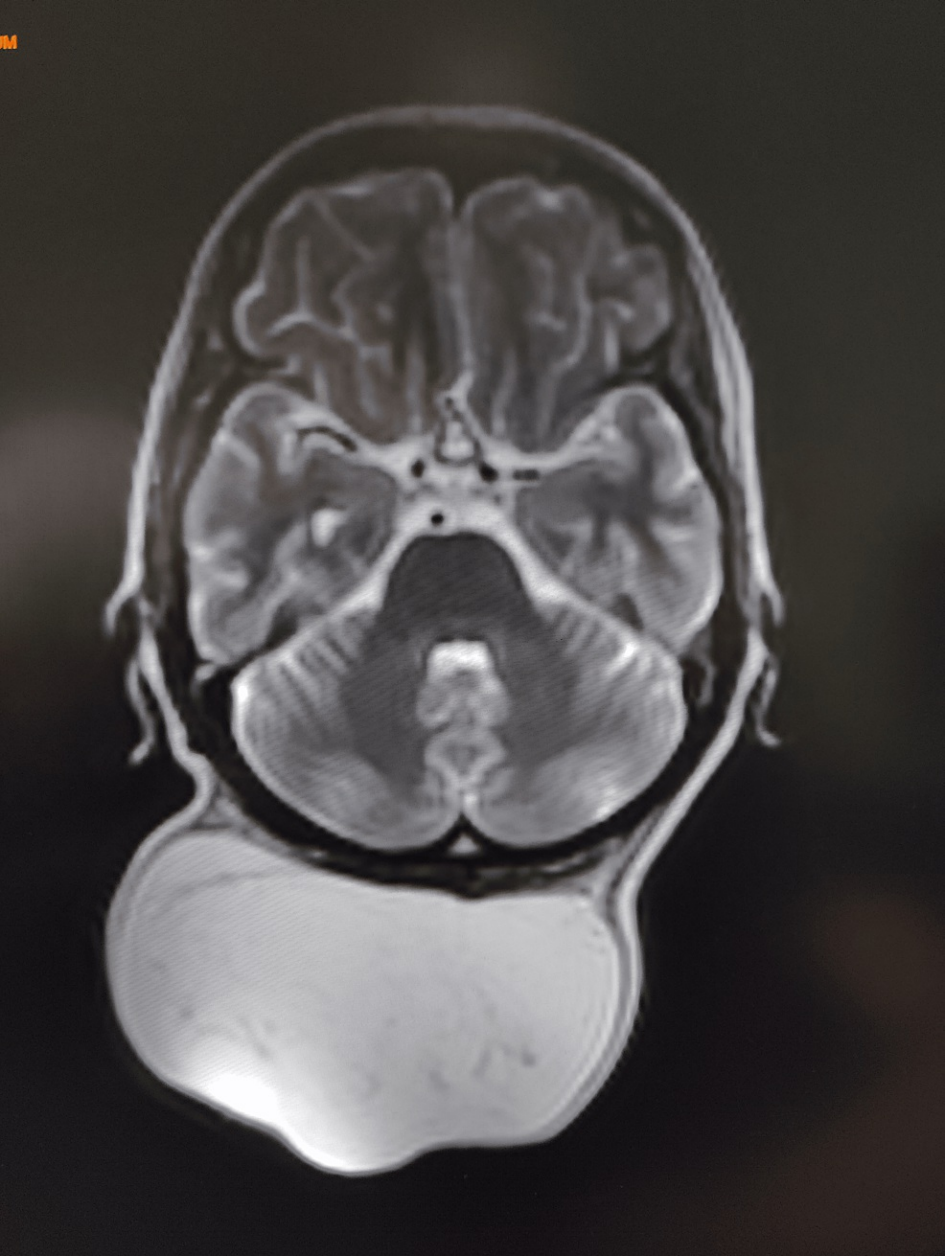
Cervical MRI image showing the giant lipoma in the cervical occipital area.

Cervical ultrasound showed a large nodular image located posteriorly in the cervical region, subcutaneous, measuring 12x10x4.8 cm, with a fatty echostructure containing trabeculations without notable hypervascularization and no associated anomalies. Cervical MRI revealed a large, encapsulated, exophytic soft tissue formation in the occipital region, roughly oval with lobulated contours, well-defined, showing hyperintense signals on T1 and T2, which disappear on the STIR sequence. The mass contained fine septa, was not enhanced after Gadolinium injection, and measured 115x98x67 mm, with no evident tissue or calcification components within the described formation. It was situated on the occipital bone and the neck's muscular structures without signs of invasion (Figure 2).

**Figure 3:**
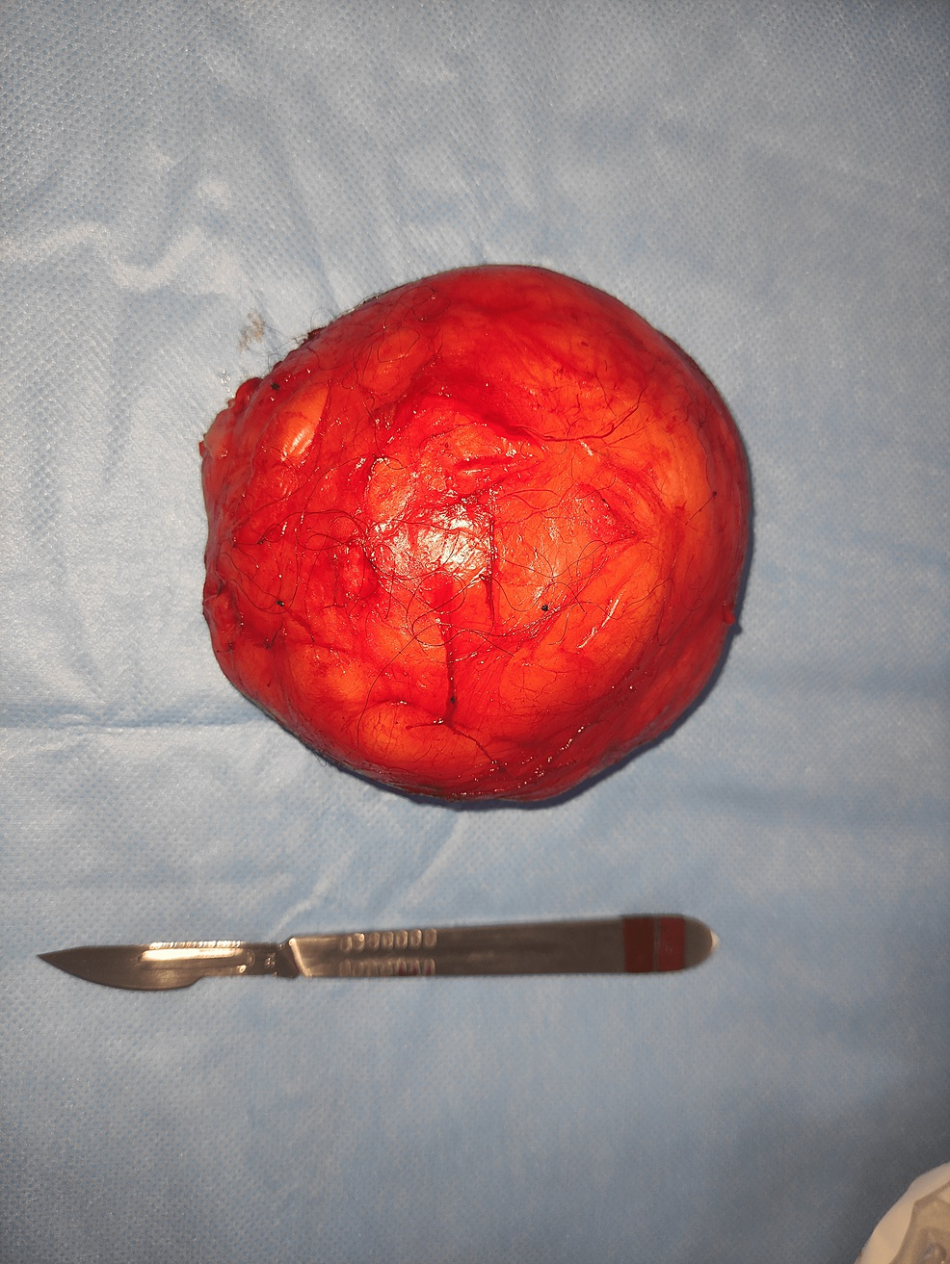
Macroscopic image of the excised lipoma

**Figure 4:**
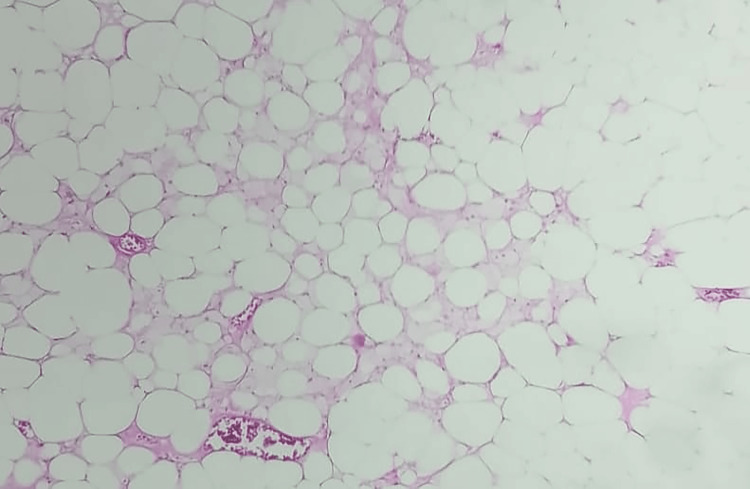
Microscopic analysis showing benign tumor proliferation made up of mature fat cells with no cytonuclear atypia.

The patient underwent surgical enucleation of the mass under general anesthesia (Figure 3). Postoperative recovery was uneventful. Pathological results showed a benign tumor proliferation of adipose tissue composed of vacuolated cytoplasmic adipocytes with peripheral nuclei arranged in lobules, separated by fibrous septa (Figure 4). Extensive sampling showed no lipoblasts, ruling out liposarcoma. Follow-up over two years was unremarkable.

## Discussion

A lipoma is a non-cancerous tumor that infrequently appears in the head and neck area. It originates from mesenchymal tissue. These tumors are usually diagnosed in individuals between the ages of 50 and 60. Most lipomas are slow-growing, and generally firm [1,4].

Most lipomas pose no diagnostic difficulties. However, in the presence of large masses (>10 cm) or rapid growth, especially in the head and neck region, a malignant tumor should be considered. Rarely, lipomas may be initially malignant or become so over time [5].

The pathogenesis of lipomas is poorly understood. Genetic, endocrine, and traumatic theories are commonly considered [6]. Understanding the mechanism behind the uncontrolled growth of these lipomas remains elusive. The skin of the posterior cervical region is particularly firm and thick, and the neck is one of the most mobile parts of the body. Blunt trauma invariably causes inflammation in the soft tissues [7].

In general, a physical examination is sufficient for diagnosing lipomas. However, radiological imaging is necessary to evaluate the characteristics of the lipoma and plan for surgery [1]. Although ultrasonography serves as one of the initial imaging modalities, it is difficult to stage the tumor based solely on this method. These masses usually present as hypoechoic homogeneous lesions [8]. CT scans and MRI are the most effective methods for establishing a definitive and differential diagnosis of cervicofacial lipomas. Classic lipomas are composed of mature adipose tissue and appear on imaging as homogeneous hypoattenuated masses, like subcutaneous fat. The shape of these lesions can vary with depth due to compression and deformation by adjacent body structures [9]. Given its superior soft tissue contrast resolution, MRI has become the preferred imaging modality for precisely determining the location and extent of the tumor. Additionally, MRI provides the best visualization of the planes between the lipoma, muscle, and blood vessels [9].

The treatment of choice is complete excision, typically straightforward due to the well-defined pseudocapsule. Confirmation of the diagnosis is through an anatomopathological study. Macroscopically, lipomas appear as soft, yellowish, shiny, smooth, mobile masses, encapsulated, and possibly with fine partitions. Microscopically, the lesions show lobular growth of mature adipocytes with demarcated borders, a fibrous capsule, and a central vacuole [5]. Progression is generally good, with recurrence observed in 4 to 5% of cases, particularly in infiltrating or deep lipomas [5].

It is essential to distinguish giant lipomas from liposarcomas, malignant fibrous histiocytomas, or other benign soft tissue lesions such as old muscle ruptures, epidermoid cysts, angiolipomas, deep haemangiomas, and diffuse lipoblastomatosis [3]. 

## Conclusions

Lipoma is a benign neoplasm histologically derived from mesodermal origin and lipomatous tissue; this neoplasm is rarely seen in the head and neck region. The overwhelming majority of lipomas are benign and have relatively slow growth, so there should be no difficulties in their diagnosis; however, one should note that some large or rapidly growing lesions in the head and neck area may raise suspicion for malignancy or a malignant transformation; in such cases, a CT or MRI scan should be performed to better define the lesion. The treatment modalities include wide surgical resection of the tumor. Knowledge of the giant lipoma pathological variant and how it may differ from other malignant or benign soft tissue tumors is significant.
